# SETD1A drives stemness by reprogramming the epigenetic landscape in hepatocellular carcinoma stem cells

**DOI:** 10.1172/jci.insight.168375

**Published:** 2023-09-22

**Authors:** Jianxu Chen, Zhijie Xu, Hongbin Huang, Yao Tang, Hong Shan, Fei Xiao

**Affiliations:** 1 Guangdong Provincial Engineering Research Center of Molecular Imaging,; 2 Department of Infectious Diseases,; 3 Department of Gastroenterology, and; 4 Center for Interventional Medicine, the Fifth Affiliated Hospital, Sun Yat-Sen University, Zhuhai, China.

**Keywords:** Hepatology, Oncology, Epigenetics, Liver cancer, Oncogenes

## Abstract

Cancer stem cells (CSCs) are responsible for tumor progression and recurrence. However, the mechanisms regulating hepatocellular carcinoma (HCC) stemness remain unclear. Applying a genome-scale CRISPR knockout screen, we identified that the H3K4 methyltransferase SETD1A and other members of Trithorax group proteins drive cancer stemness in HCC. SET domain containing 1A (SETD1A) was positively correlated with poor clinical outcome in patients with HCC. Combination of SETD1A and serum alpha fetoprotein substantially improved the accuracy of predicting HCC relapse. Mechanistically, SETD1A mediates transcriptional activation of various histone-modifying enzymes, facilitates deposition of trimethylated H3K4 (H3K4me3) and H3K27me3, and activates oncogenic enhancers and super-enhancers, leading to activation of oncogenes and inactivation of tumor suppressor genes simultaneously in liver CSCs. In addition, SETD1A cooperates with polyadenylate-binding protein cytoplasmic 1 to regulate H3K4me3 modification on oncogenes. Our data pinpoint SETD1A as a key epigenetic regulator driving HCC stemness and progression, highlighting the potential of SETD1A as a candidate target for HCC intervention and therapy.

## Graphical abstract

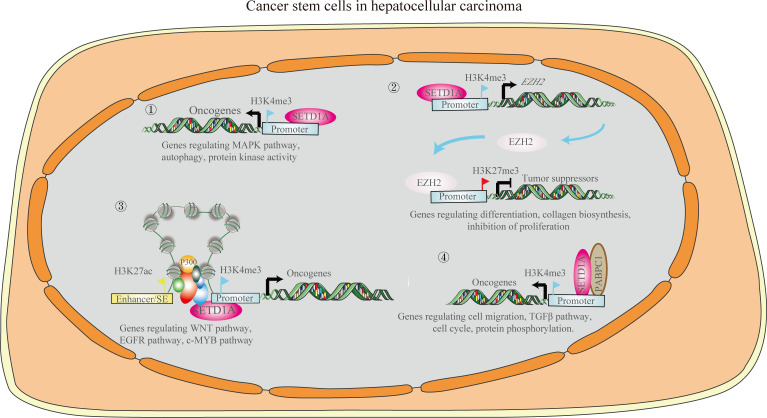

## Introduction

Hepatocellular carcinoma (HCC) is one of the leading causes of cancer-related death and displays high level of heterogeneity, which limits the efficacy of clinical treatment. The heterogeneity of HCC has been reported to be driven by cancer stem cells (CSCs), which are a subpopulation of tumor cells with self-renewal capacity and differentiation potential (1). Since John Dick isolated leukemia stem cells in 1994, accumulating evidence has shown that CSCs are responsible for cancer drug resistance, relapse, and metastasis. Therefore, CSCs represent promising targets for cancer therapy (2). Understanding determinants and the regulatory mechanism of CSCs’ self-renewal is important in developing CSC-targeted therapy.

Cell surface markers CD24, CD133, CD90, EpCAM, and CD13 are frequently used to identify and isolate liver CSCs (3). However, liver CSCs expressing different markers have discrete characteristics and tumor-initiating capacity. For example, EpCAM^+^ liver CSCs have features of epithelial cells and are associated with rapid growth, while CD90^+^ liver CSCs present features of vascular endothelial cells and with high incidence of distant organ metastasis (4). Compared with CD24^+^ liver CSCs and CD133^+^ liver CSCs, CD24^+^CD133^+^ liver CSCs have a stronger tumor-initiating capacity. As few as 10 CD24^+^CD133^+^ liver CSCs could initiate tumorigenicity in NOD/SCID mice, and patients with CD24^+^CD133^+^ liver CSCs have worse clinical outcome, revealing the critical role of CD24^+^CD133^+^ CSCs in HCC tumorigenesis (5).

High-throughput CRISPR screening is a powerful tool for discovering key regulators in cancer development, progression, and resistance, with the advantage of low-noise, minimal off-target effects and consistently high efficiency over RNAi-based genetic screening (6). Using kinome CRISPR knockout library, kinases CDK7, CDK12, and CDC7 have been identified to regulate HCC progression (7). Here, we established a genome-wide CRISPR knockout screening in CD24^+^CD133^+^ liver CSCs and identified the H3K4 methyltransferase SET domain containing 1A (SETD1A) as a critical diver for HCC stemness and progression, providing a therapeutic target for HCC treatment.

## Results

### A genome-wide CRISPR screening for factors contributing to HCC stemness.

**Figure 1:**
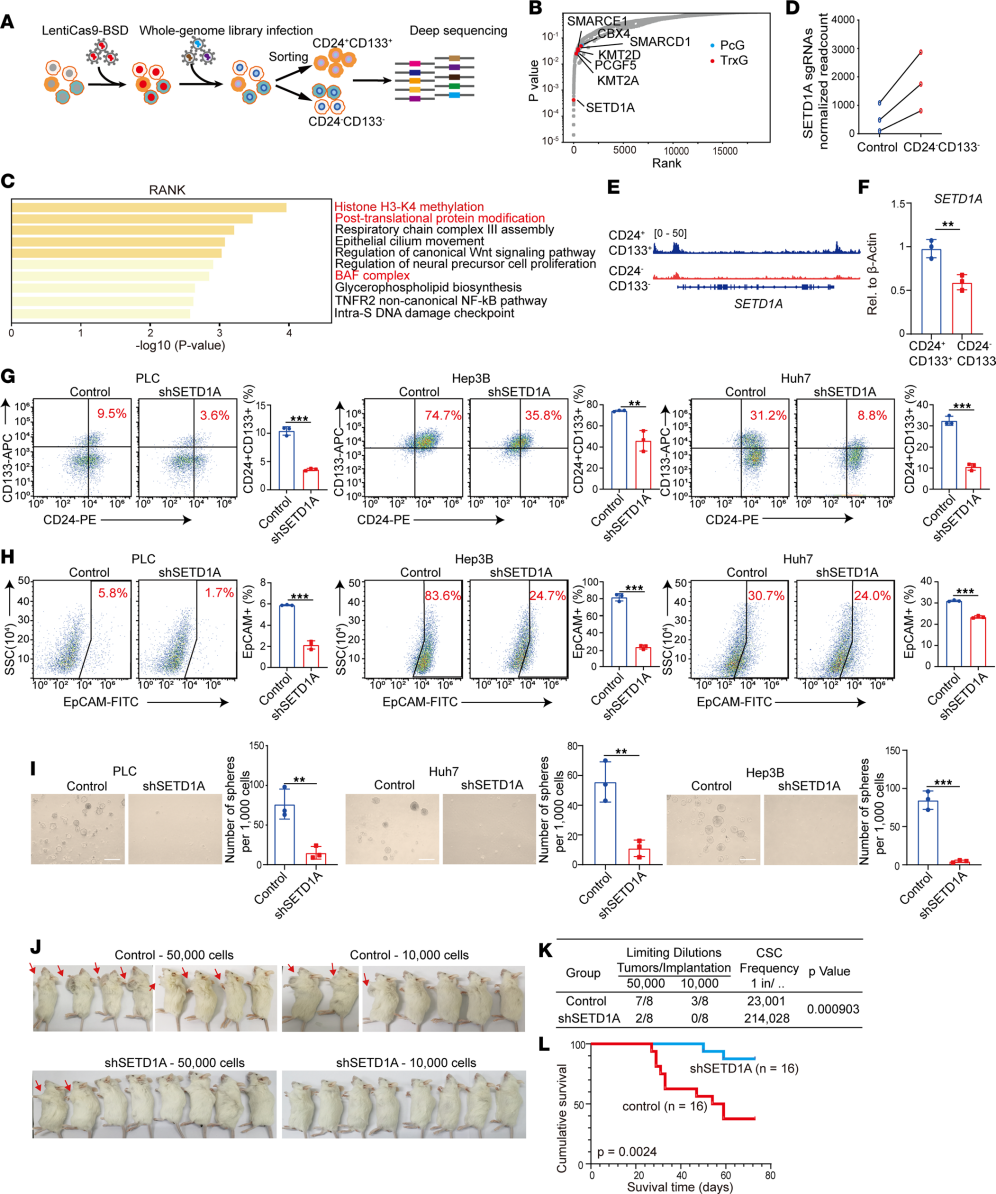
SETD1A promotes HCC stemness in vitro and in vivo. (**A**) Schematic outline of CRISPR/Cas9 knockout screen. (**B**) A scatter plot of the gRNA distribution from the GeCKO screen. The TrxG proteins are labeled in red, and the PcG proteins are labeled in blue. (**C**) GO enrichment analysis of the hits promoting liver CSCs’ expansion. (**D**) Enrichment of the gRNAs targeting SETD1A in the CD24^–^CD133^–^ non-CSCs. (**E**) ATAC-Seq analysis of the accessibility of SETD1A locus in the CD24^+^CD133^+^ CSCs and CD24^–^CD133^–^ non-CSCs. (**F**) qRT-PCR analysis of SETD1A expression in CD24^+^CD133^+^ CSCs and CD24^–^CD133^–^ non-CSCs (*n* = 3). Comparison of the proportion of CD24^+^CD133^+^ CSCs (**G**) as well as EpCAM^+^ CSCs (**H**) in scramble control and SETD1A-knockdown HCC cells using flow cytometry (*n* = 3). (**I**) The spheroid formation assays showing the role of SETD1A in HCC stemness in vitro (*n* = 3). Scale bar represents 500 μm. (**J**) The images of tumors’ formation in NOD/SCID mice injected subcutaneously with the scramble control and shSETD1A HCC cells. (**K**) Extreme Limiting Dilution Analysis for comparing the scramble control group and shSETD1A group. (**L**) Overall survival curves of mice transplanted with the scramble control and shSETD1A HCC cells. Data are presented as mean ± SEM. Statistical analysis was performed by unpaired 2-tailed Student’s *t* test. **P < 0.01, and ***P < 0.001.

We used a pooled genome-wide CRISPR/Cas9 knockout library (GeCKO v2) consisting of 58,028 gRNAs targeting 19,009 genes (3 guide RNAs [gRNAs] per gene) to investigate genes contributing to stemness of CD24^+^CD133^+^ HCC in the PLC cell line (Figure 1A and Supplemental Figure 1A; supplemental material available online with this article; https://doi.org/10.1172/jci.insight.168375DS1). We ranked sgRNAs that were enriched in CD24^–^CD133^–^ non-CSCs compared with the nonsorted library cell population using MAGeCK robust ranking algorithm (Figure 1B). We identified 504 genes contributing to HCC stemness, including ABCG1, CDK16, and MYCT1, which have been reported to drive HCC stemness (Supplemental Figure 1, B and C) (8–10). Interestingly, we found several top hits belonging to Trithorax group (TrxG) and Polycomb group (PcG) proteins, including KMT2A (also known as MLL/MLL1), KMT2D (also known as MLL2/MLL4), SETD1A, SMARCD1, SMARCE1, PCGF5, and CBX4 (Supplemental Figure 1D). TrxG and PcG proteins activate oncogenes’ transcription and inhibit transcription of tumor suppressors through regulating histone modification and higher order chromatin structure (11). PcG protein CBX4 has been reported to promote HCC stemness and increase sorafenib resistance in advanced HCC (12). Another PcG protein, PCGF5, is also associated with CSCs’ expansion (13). TrxG proteins SMARCD1 and SMARCE1 have been reported to promote tumor progression (14, 15). KMT2A and KMT2D are the targets of mutant P53^R249S^, which is the most common missense mutation of p53 (16). In addition, KMT2A is essential for HGF/MET signaling–induced HCC metastasis (17). Gene ontology (GO) enrichment analysis of the 504 hits indicated that GO terms of H3K4 methylation, posttranslation protein modification, and SWI/SNF complex were prominently enriched, revealing these epigenetic regulators, especially TrxG proteins, play a core role in liver CSCs’ expansion. Other prominently enriched GO terms included WNT pathway (18) and noncanonical NF-κB pathway (19), which also have been proven to promote HCC stemness (Figure 1C). Collectively, our CRISPR screening identified TrxG and PcG proteins as key factors contributing to HCC stemness.

### SETD1A promotes HCC stemness in vitro and in vivo.

Histone methyltransferase SETD1A, which is the catalytic subunit of the SET1/COMPASS complex containing WDR5, ASH2L, RBBP5, DPY30, SETD1B, HCF1, WDR82, and CFP1, was among the top-ranking genes in the screen analysis. gRNAs targeting SETD1A were significantly enriched in non-CSCs (Figure 1D). It has been reported that SETD1A is associated with cell differentiation, development, and tumor progression (20). A previous study using hepatocyte-like cells differentiated from human induced pluripotent stem cells (iPSCs) showed that SETD1A incorporated with CUDR to trigger the malignant transformation of hepatocytes. Overexpression of SETD1A alone failed to promote malignant transformation (21). However, our data revealed SETD1A as an independent regulator of HCC stemness. The discrepancy between our results and the previous finding could be explained by the iPSC-derived hepatocyte-like cells used in the previous study, which could not fully display functional characteristics of hepatic stem/progenitor cells (3, 22). To further investigate the role of SETD1A in HCC stemness, we first used ATAC-Seq assay to determine its chromatin states in liver CSCs and found its promoter region was more accessible in CD24^+^CD133^+^ liver CSCs than in CD24^–^CD133^–^ non-CSCs (Figure 1E). Quantitative real-time PCR (qRT-PCR) assay also showed that *SETD1A* was significantly upregulated in CD24^+^CD133^+^ HCC CSCs compared with CD24^–^CD133^–^ non-CSCs, verifying the result of ATAC-Seq assay (Figure 1F). These results demonstrated SETD1A was transcriptionally activated in liver CSCs.

To validate the function of SETD1A in HCC stemness, we established stable HCC cell lines of PLC, Huh-7, and Hep3B with shRNA targeting SETD1A, then confirmed its knockdown by Western blotting and qRT-PCR assay (Supplemental Figure 1, E and F). Notably, SETD1A knockdown significantly decreased the proportion of CD24^+^CD133^+^ liver CSCs (Figure 1G and Supplemental Figure 1G). Meanwhile, we observed that SETD1A knockdown significantly reduced the proportion of EpCAM^+^ liver CSCs (Figure 1H). Spheroid formation assays revealed SETD1A knockdown effectively inhibited spheroid formation (Figure 1I). Moreover, we assessed the tumorigenicity of serial dilutions of SETD1A-knockdown HCC cells in NOD/SCID mice. As shown in Figure 1, J and K, SETD1A knockdown significantly reduced tumor-initiating capacity and CSCs’ frequency. Kaplan-Meier survival assay showed that the mice transplanted with SETD1A-knockdown HCC cells had significantly longer survival time than those transplanted with scramble control HCC cells (Figure 1L). Taken together, we demonstrated that SETD1A promoted HCC stemness in vitro and in vivo. In addition, we found SETD1A significantly promoted HCC cell proliferation, invasion, migration, and sorafenib resistance and induced epithelial-mesenchymal transition (EMT) (Supplemental Figure 2), suggesting SETD1A promotes HCC progression.

### SETD1A is upregulated in HCC tissues and associated with poor clinical outcome in patients with HCC.

**Figure 2:**
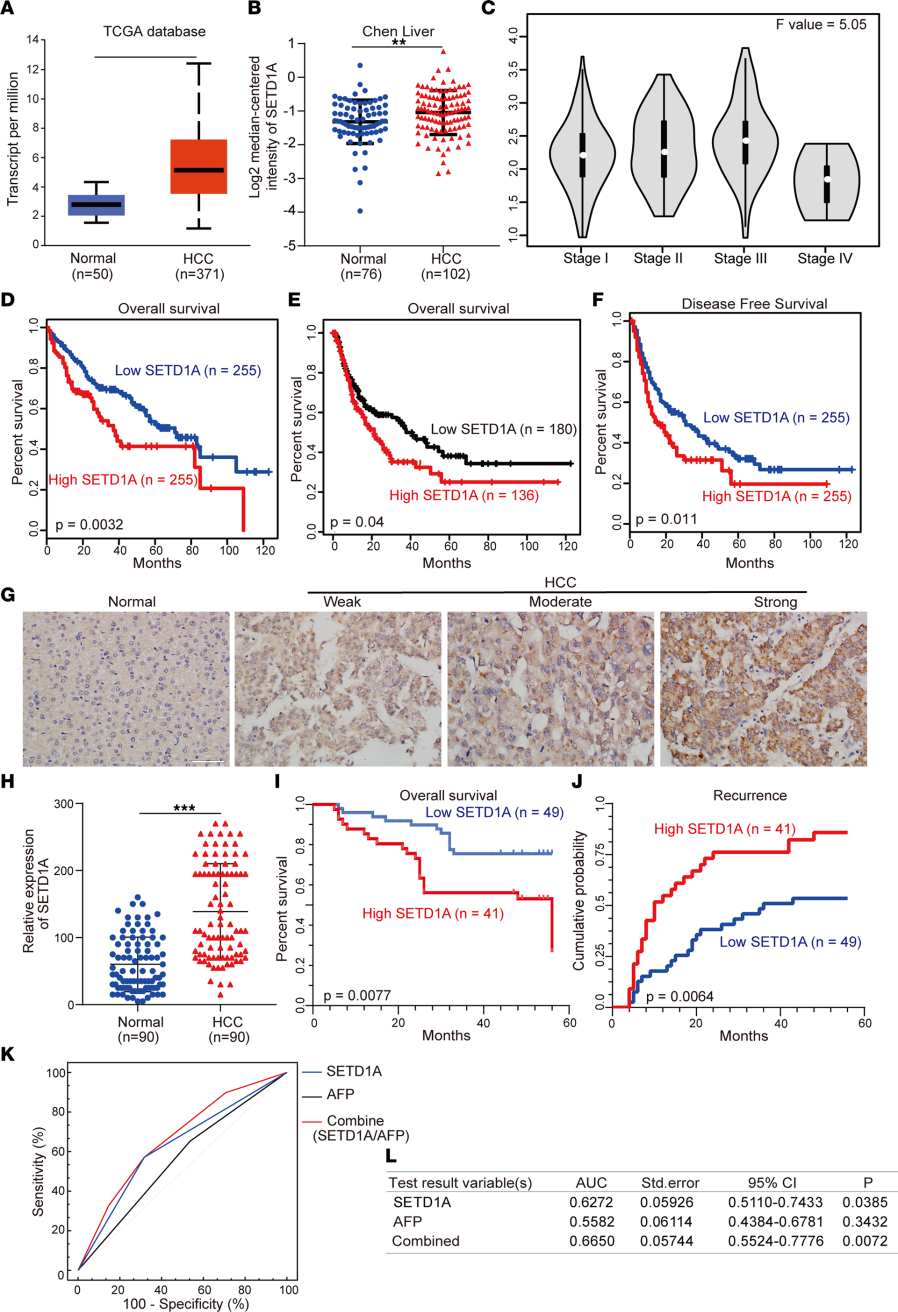
High-level expression of SETD1A is associated with poor outcome in patients with HCC. Analysis of relative expression of SETD1A in HCC samples and their matched normal samples using TCGA (**A**) and Oncomine (**B**) database. Box plots show the interquartile range (box), median (line), and minimum and maximum (whiskers). (**C**) Relative expression of SETD1A at different HCC stages. (**D** and **E**) Analysis of overall survival curves of patients with high and low SETD1A expression levels using TCGA (**D**) and Kaplan-Meier Plotter (**E**) databases. (**F**) Analysis of disease-free survival curves in HCC patients with high or low SETD1A expression using TCGA database. (**G**) IHC analysis of SETD1A expression in human HCC tissue microarrays. Scale bar represents 60 μm. (**H**) Analysis of relative expression of SETD1A in HCC samples and their matched normal samples using IHC. (**I**) Analysis of overall survival curves of HCC patients with high and low SETD1A expression levels. (**J**) Analysis of recurrence curves of HCC patients with high and low SETD1A expression levels. (**K** and **L**) ROC analysis for evaluating the association between SETD1A and HCC recurrence. **P < 0.01, and ***P < 0.001.

To investigate the clinical significance of SETD1A in HCC, we determined SETD1A expression in HCC tissues using The Cancer Genome Atlas (TCGA) database and Oncomine database. We found SETD1A was significantly upregulated in HCC tissues compared with their matched adjacent normal liver tissues (Figure 2, A and B). Its expression was associated with advanced stages of HCC (Figure 2C). Then, we performed Kaplan-Meier survival assay using TCGA and Kaplan-Meier Plotter databases and found the overall survival and disease-free survival of patients with high SETD1A expression was significantly shorter than those with low SETD1A expression (Figure 2, D–F). Immunohistochemical (IHC) analysis of 90 matched pairs of HCC and adjacent normal liver tissues using IHC staining verified high expression of SETD1A in HCC tissue (Figure 2, G and H). SETD1A expression was positively correlated with tumor size, tumor encapsulation, and tumor recurrence (Table 1), which was further validated by univariate and multivariate Cox proportional hazard regression analyses (Table 2). Higher SETD1A expression was associated with shorter survival times and higher recurrence rate (Figure 2, I and J). Using receiver operator characteristic (ROC) analysis, we showed that SETD1A exhibited better performance than serum alpha fetoprotein (AFP) in prediction of HCC relapse (23). Combination of SETD1A and serum AFP significantly improved the accuracy in prediction of HCC relapse (Figure 2, K and L). Taken together, these findings indicated that high SETD1A expression was associated with poor outcome and relapse in patients with HCC.

### SETD1A promotes HCC stemness and progression by directly transcriptionally activating histone-modifying enzymes.

**Figure 3:**
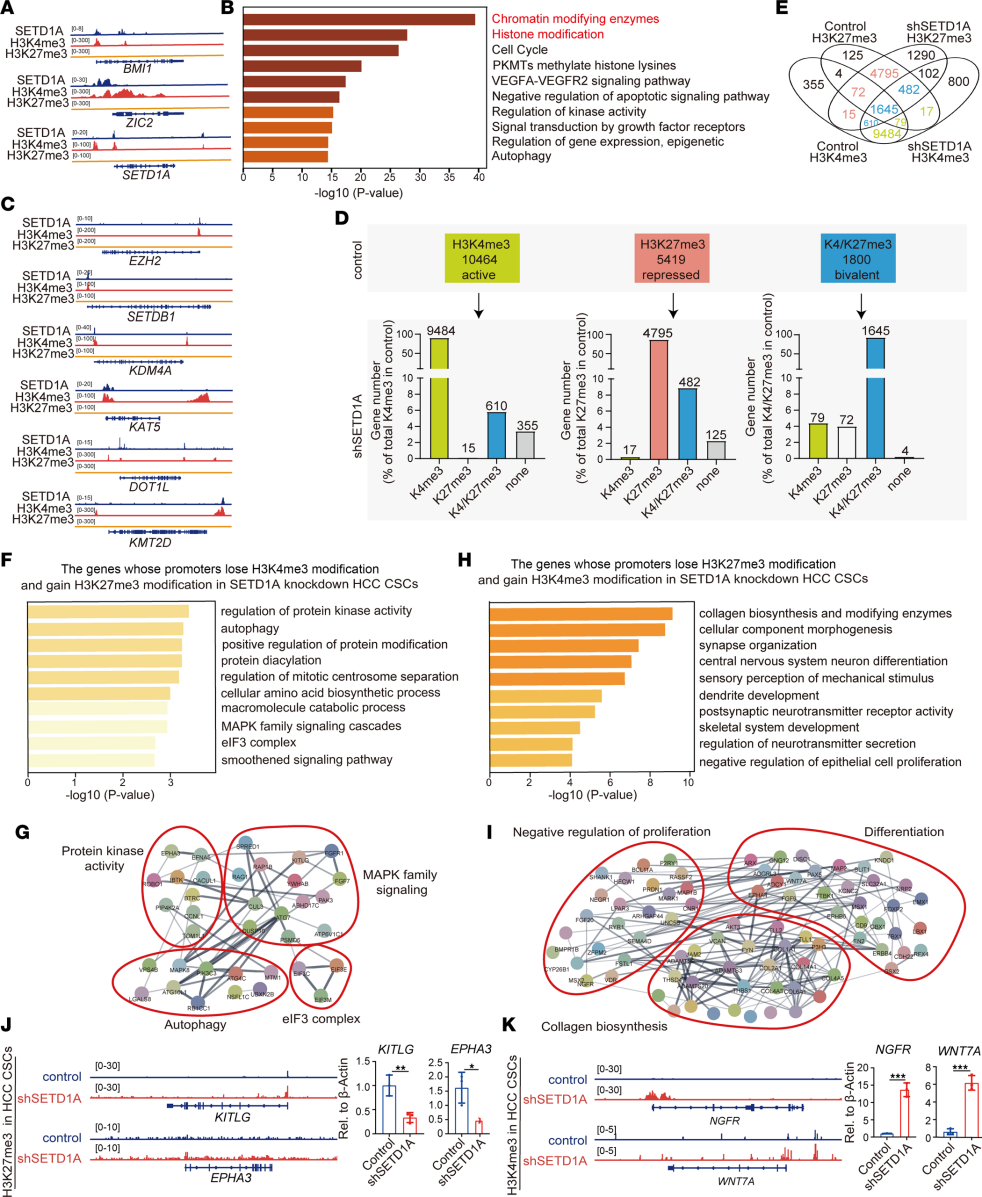
SETD1A promotes HCC stemness and progression by regulating histone modification. (**A**) Representative SETD1A, H3K4me3, and H3K27me3 CUT&Tag profiles in CD24^+^CD133^+^ CSCs at *BMI1*, *ZIC2*, *SETD1A*, and *PDK4* loci. (**B**) GO enrichment analysis of SETD1A-regulated genes in CD24^+^CD133^+^ CSCs. (**C**) Representative SETD1A, H3K4me3, and H3K27me3 CUT&Tag profiles in CD24^+^CD133^+^ CSCs at *EZ* *H2*, *SETD1B*, *KDM4A*, *KAT5*, *DOT1L*, and *KMT2D* loci. (**D**) The changes of H3K4me3-marked genes (indicated as K4me3), H3K37me3-marked genes (indicated as K27me3), and bivalent genes (indicated as K4/K27me3) resulting from SETD1A knockdown. (**E**) Venn diagram of the binding sites of H3K4me3 and H3K27me3 in control and SETD1A-knockdown HCC. (**F**) GO enrichment analysis of the genes losing H3K4me3 and gaining H3K27me3 resulting from SETD1A knockdown. (**G**) PPI network analysis of the regulators losing H3K4me3 and gaining H3K27me3 upon SETD1A knockdown. (**H**) GO enrichment analysis for the genes gaining H3K4me3 and losing H3K27me3 upon SETD1A knockdown. (**I**) PPI network analysis of the regulators gaining H3K4me3 and losing H3K27me3 upon SETD1A knockdown. (**J**) Representative H3K27me3 CUT&Tag profiles in the control and SETD1A-knockdown CD24^+^CD133^+^ CSCs at *KITLG* and *EPHA3* loci. The expression of KITLG and EPHA3 is shown on the right. (**K**) Representative H3K4me3 CUT&Tag profiles in control and SETD1A-knockdown CD24^+^CD133^+^ CSCs at *NGFR* and *WNT7A* loci. Their expression of NGFR and WNT7A is shown on the right (*n* = 3). Data are presented as mean ± SEM. Statistical analysis was performed by unpaired 2-tailed Student’s *t* test. *P < 0.05, **P < 0.01, and ***P < 0.001.

The SETD1A-generated trimethylated H3K4 (H3K4me3) modification marks the promoters of actively transcribed genes. To further determine the regulatory mechanism of SETD1A driving HCC stemness, we used CUT&Tag to investigate the genomic distributions of SETD1A and H3K4me3 in CD24^+^CD133^+^ liver CSCs. H3K27me3 modification marks the promoters of transcriptionally silent genes. To determine the transcriptional activation of genes, we also investigate the genomic distribution of H3K27me3. CUT&Tag assay showed that SETD1A and H3K4me3 co-occupied the promoters of some known drivers of HCC stemness, such as PRMT6, BMI1, SOX9, ZIC2, ANGPTL4, PDK4, and IRAK1 (24), suggesting SETD1A activated the transcription of these genes. Notably, we found that SETD1A formed a positive feedback loop with itself through binding to its own promoter (Figure 3A and Supplemental Figure 3A). Importantly, the GO analysis of the top 3,000 SETD1A-regulated genes showed that chromatin-modifying enzymes and histone modification were the most prominently enriched (Figure 3B). Alterations of chromatin modification are linked to dysregulated expression of genes critical for tumorigenesis and development (25). SETD1A and H3K4me3 co-occupied the promoters of many histone methyltransferases, histone demethylases, and histone acetyltransferases, including KMT2A, KMT2D, H3K27me3 methyltransferase EZH2, H3K9 methyltransferase SETDB1, H3K9me3 and H3K36me3 demethylase KDM4A, H3K9me3 methyltransferase SUV39H2 and SUV39H1, H3K79 methyltransferase DOT1L, H3K20me1/me2 methyltransferase KMT5C, lysine acetyltransferase KAT6A, KAT5, KAT7, and KAT8 (Figure 3C and Supplemental Figure 3B). In addition, the promoters of these genes are not bound by H3K27me3, suggesting these genes are transcriptional activation in liver CSCs. Among them, EZH2, SETD1B, KDM4A, DOTL1, SUV39H1, KAT7, KAT8, and KAT5 have been reported to promote HCC stemness, HCC growth, EMT, and metastasis (26). These results reveled that SETD1A promotes the HCC stemness through depositing H3K4me3 on the promoters of various histone modifiers to promote their transcription, highlighting the core role of SETD1A in epigenetic regulation of HCC stemness.

### SETD1A knockdown remodels the chromatin modification states of H3K4me3 and H3K27me3.

We found that SETD1A and H3K4me3 co-occupied the promoters of EZH2 (Figure 3C). Knockdown of SETD1A inhibited EZH2 expression (Supplemental Figure 3C), suggesting SETD1A transcriptionally activates EZH2. In addition, EZH2 catalyzes H3K27me3 and promotes HCC stemness and progression (27). These findings suggested that SETD1A regulates H3K27me3 modification. Therefore, we speculate that SETD1A may promote HCC stemness through depositing H3K4me3 on the promoters of oncogenes and H3K27me3 on the promoters the tumor suppressor genes. Next, we determined the genome-wide profiles of H3K4me3 and H3K27me3 in CD24^+^CD133^+^ liver CSCs and SETD1A-knockdown CD24^+^CD133^+^ liver CSCs. A total of 10,464 H3K4me3-marked genes and 5,419 H3K27me3-marked genes were detected in CD24^+^CD133^+^ HCC CSCs. Meanwhile, a total of 10,380 H3K4me3-marked genes and 6,172 H3K27me3-marked genes were detected in SETD1A-knockdown CD24^+^CD133^+^ liver CSCs (Supplemental Figure 3D). In H3K4me3-marked genes, SETD1A knockdown resulted in 15 (~0.15%) genes gaining H3K27me3, 610 (~5.8%) bivalent genes, and 355 (~3.4%) genes losing H3K4me3 mark. In H3K27me3-marked genes, SETD1A knockdown resulted in 17 (~0.3%) genes gaining H3K4me3 and 482 (~8.9%) bivalent genes. In 1,800 bivalent genes, SETD1A knockdown resulted in 79 genes (4.4%) losing H3K27me3 and 72 genes (~4.0%) losing H3K4me3 (Figure 3, D and E). On the pathway level, we determined the impact of SETD1A knockdown on the effect of H3K4me3 and H3K27me3 modification dynamics in HCC stemness using GO analysis. GO terms associated with protein kinase activity, autophagy, MAPK signaling, and eIF3 complex were significantly enriched among the genes gaining H3K27me3 and losing H3K4me3 (Figure 3F). Analysis of protein–protein interactions (PPIs) of these genes revealed 4 functional modules including MAPK signaling pathway, autophagy, eIF3 family, and protein kinases (Figure 3, G and J), all of which have been reported to play a crucial role in HCC stemness as well as cancer progression (28). To determine the function of the genes losing H3K27me3 and gaining H3K4me3 upon SETD1A knockdown, we performed GO and PPI network analysis and found that these genes were involved in collagen biosynthesis, differentiation, and negative regulation of proliferation (Figure 3, H, I, and K). Then we used qRT-PCR and Western blot to verify above findings and found SETD1A knockdown significantly inhibited genes associated with MAPK pathway, autophagy, and protein kinase activity, such as *RAG1*, *ATG7*, *MAPK8*, *MTM1*, *CCNL1*, and *EFNA5*. Meanwhile, SETD1A knockdown significantly upregulated genes associated with negative regulation of proliferation, differentiation, and collagen biosynthesis, such as *MSX2*, *ZFPM2*, *FGF9*, *PAX5*, *COL1A1*, *TLL1*, and *TLL2* (Supplemental Figure 4A). Western blotting also showed SETD1A knockdown promoted collagen I expression (Supplemental Figure 4B). Taken together, these results suggested that SETD1A increased promoter activity of oncogenes and inhibited promoter activity of tumor suppressor genes to drive HCC stemness.

### SETD1A drives HCC stemness by mediating H3K27ac deposition.

**Figure 4:**
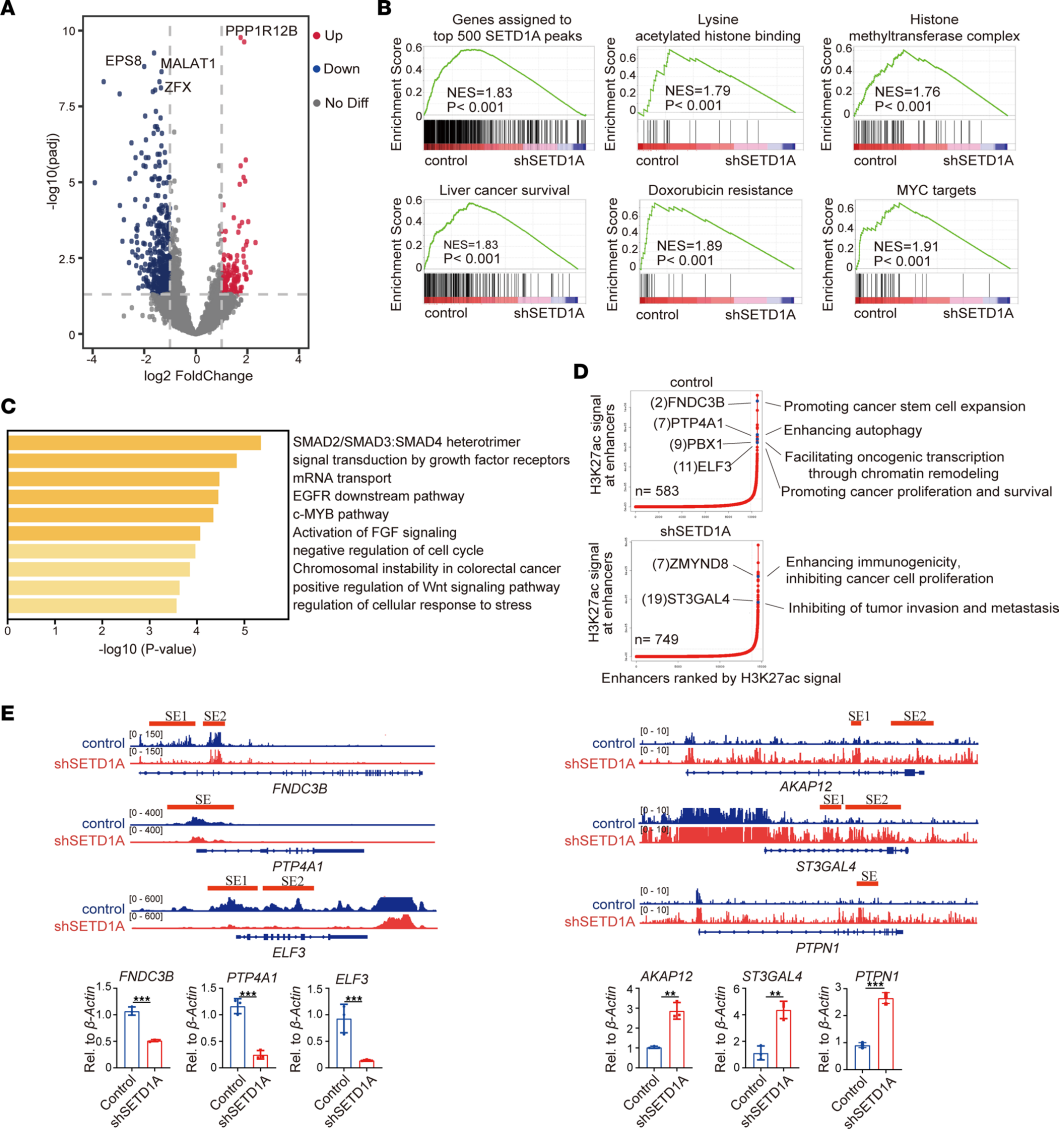
SETD1A promotes the activity of oncogenic enhancers and super-enhancers. (**A**) Volcano plots illustrating distribution of the enhancer-associated genes in the SETD1A-knockdown versus control CD24^+^CD133^+^ CSCs. Genes losing H3K27ac are marked in blue. Genes gaining H3K27ac are marked in red. (**B**) Leading-edge analysis of the enrichment of genes losing H3K27ac in the SETD1A-knockdown versus control CD24^+^CD133^+^ CSCs based on GSEA. (**C**) GO enrichment analysis of the genes losing H3K27ac in SETD1A-knockdown versus control in CD24^+^CD133^+^ CSCs. (**D**) Distribution of H3K27ac signal across enhancers in the control CD24^+^CD133^+^ CSCs and SETD1A-knockdown CD24^+^CD133^+^ CSCs. Prominent genes associated with SEs are highlighted with their respective SE ranks and roles in tumor initiation and progression. (**E**) Representative H3K27ac CUT&Tag profiles in the control and SETD1A-knockdown CD24^+^CD133^+^ CSCs at *FNDC3B*, *PTP4A1*, *ELF3*, *AKAP12*, *ST3GAL4*, and *PTPN1* locus (top). The expression of *FNDC3B*, *PTP4A1*, *ELF3*, *AKAP12*, *ST3GAL4*, and *PTPN1* is shown on the bottom (*n* = 3). Data are presented as mean ± SEM. Statistical analysis was performed by unpaired 2-tailed Student’s *t* test. **P < 0.01, and ***P < 0.001.

Enhancer malfunction drives the aberrant regulation of oncogenes in cancer. H3K27ac, which marks active enhancers, is directly blocked by H3K27me3 and has a synergistic effect with H3K4me3 on tumor progression (29, 30). Thus, we determined the H3K27ac-enriched enhancer regions in CD24^+^CD133^+^ CSCs and SETD1A-knockdown CD24^+^CD133^+^ CSCs, and we found that SETD1A knockdown resulted in the change of H3K27ac distribution. We observed loss of H3K27ac mark in the enhancers of 442 genes, including long noncoding RNA (lncRNA) *MALAT1* and oncogenes *ZFX* and *EPS8*, as well as gain of H3K27ac mark in the enhancers of 134 genes, including the tumor suppressor *PPP1R12B* (31, 32) (Figure 4A). Gene set enrichment analysis (GSEA) of the genes associated with loss of H3K27ac revealed that SETD1A knockdown resulted in loss of SETD1A binding sites signature, histone methyltransferase complex signature, and liver cancer growth–associated, doxorubicin resistance, and MYC target genes, all of which have been reported to be associated with CSCs’ stemness, therapeutic resistance, and tumor progression (33) (Figure 4B). In addition, GO analysis of the genes associated with loss of H3K27ac were prominently enriched in signaling pathways related to CSCs’ stemness, tumorigenesis, and tumor growth (Figure 4C). Together, these findings suggested that SETD1A increased activity of oncogenic enhancers to promote HCC stemness and progression.

Super-enhancers (SEs) that recruit a great number of transcription factors and cofactors to confer strong transcriptional regulation have been proven to play an essential role in CSCs’ self-renewal and tumor progression through increasing transcriptional activity of oncogenes (34). To determine whether SETD1A regulates SEs, we annotated 583 SEs and 749 SEs in CD24^+^CD133^+^ liver CSCs and SETD1A-knockdown CD24^+^CD133^+^ liver CSCs, respectively, and observed that SE-associated genes, such as *FNDC3B*, *PTP4A1*, *PBX1* (35), and *ELF3* (28), promote CSCs’ self-renewal and tumor progression in CD24^+^CD133^+^ liver CSCs (Figure 4D). SETD1A knockdown changed the landscape of SEs, resulting in activation of the transcription of SE-driven tumor suppressors, such as *ST3GAL4*, *AKAP12*, *PTPN1*, *AQP9*, and *ANKRD11* (Figure 4E). Overall, these results suggested that SETD1A promoted HCC stemness by increasing oncogenic activity of enhancers and SEs.

### SETD1A drives HCC stemness via interacting with polyadenylate-binding protein cytoplasmic 1 to regulate H3K4me3 modification.

**Figure 5:**
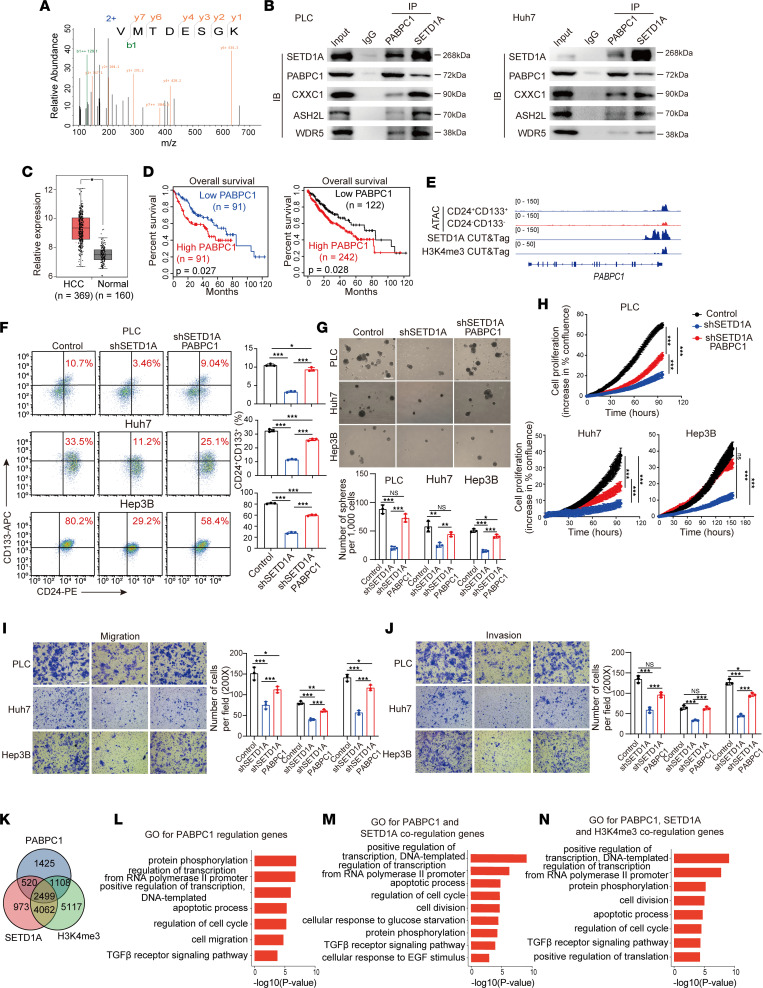
SETD1A cooperates with PABPC1 to promote HCC stemness and progression. (**A**) MS identification of PABPC1 as an interaction protein of SETD1A. (**B**) Western blots of endogenous co-IPs for PABPC1, SETD1A, CXXC1, ASH2L, and WDR5 in PLC and Huh7 cells. (**C**) Analysis of relative expression of PABPC1 in HCC samples and their matched normal samples using TCGA database. (**D**) Analysis of overall survival curves of patients with high and low PABPC1 expression levels using TCGA database. (**E**) ATAC-Seq analysis of the accessibility of *PABPC1* locus in the CD24^+^CD133^+^ CSCs and CD24^–^CD133^–^ non-CSCs and the representative SETD1A CUT&Tag profiles in CD24^+^CD133^+^ CSCs at *PABPC1* locus. (**F**) Comparison of the proportion of CD24^+^CD133^+^ CSCs in SETD1A-knockdown and PABPC1-expressing SETD1A-knockdown HCC cells using flow cytometry (*n* = 3). (**G**) The spheroid formation assays showing the effect of PABPC1 overexpression on the stemness of SETD1A-knockdown HCC cells (*n* = 3). Scale bar represents 500 μm. (**H**) Cell proliferation assay for the effect of PABPC1 overexpression on the cell proliferation of SETD1A-knockdown HCC cells (*n* = 3). (**I** and **J**) Transwell assay with/without Matrigel assessing the effect of SETD1A-knockdown PABPC1 overexpression on the migration and invasion of SETD1A-knockdown HCC cells (*n* = 3). Scale bar represents 200 μm. (**K**) Venn diagram showing the extent of overlap for PABPC1-, SETD1A-, and H3K4me3-bound regions in liver CSCs. (**L**) GO analysis for biological processes of PABPC1 target genes. (**M**) GO analysis for biological processes of PABPC1 and SETD1A co-regulation genes. (**N**) GO analysis for biological processes of PABPC1, H3K4me3, and SETD1A co-regulation genes. Data are presented as mean ± SEM. Statistical analysis was performed by unpaired 2-tailed Student’s *t* test, or 2-way ANOVA was used with post hoc test. *P < 0.05, **P < 0.01, and ***P < 0.001.

Next, we used co-immunoprecipitation (co-IP) followed by mass spectrometry (MS) to identify SETD1A-interacting proteins. Notably, we found that RNA-binding protein polyadenylate-binding protein cytoplasmic 1 (PABPC1) interacted with SETD1A and its core complex proteins (Figure 5, A and B). Cytoplasmic PABPC1 has been reported to promote the progression of different types of tumors. Mechanistically, PABPC1 mainly is regulated by noncoding RNAs, such as circPTK2 in bladder cancer (36) and lncRNA *SNHG14* in HCC (37). However, the role of nuclear PABPC1 has not been investigated to our knowledge. Previous studies showed that PABPC1 interacts with AGO2 and eukaryotic initiation factor 4G in cytoplasm to regulate mRNA translation and HCC proliferation (38, 39). Its role in promoting HCC stemness has not been studied yet to our knowledge. We showed that PABPC1 was upregulated in HCC tissues (Figure 5C). HCC patients with high PABPC1 expression had shorter survival time than patients with low PABPC1 expression (Figure 5D). CUT&Tag assay showed that SETD1A directly bound to the promoter sequences of PABPC1 in CD24^+^CD133^+^ liver CSCs. ATAC-Seq data analysis revealed that PABPC1 promoter was more accessible in CD24^+^CD133^+^ liver CSCs than in non-CD24^+^CD133^+^ HCC cells (Figure 5E), suggesting that SETD1A not only interacted with PABPC1 but also regulated the transcriptional activity of PABPC1. To determine whether SETD1A promotes HCC stemness and progression through regulating PABPC1, we overexpressed PABPC1 in SETD1A-knockdown HCC cells (Supplemental Figure 4C). FACS and spheroid formation assays showed that PABPC1 overexpression partly reversed the effect of SETD1A knockdown on the percentage of CD24^+^CD133^+^ population and sphere number (Figure 5, F and G). Cell proliferation and Transwell invasion and migration assays showed PABPC1 overexpression partly reversed the effect of SETD1A knockdown on HCC proliferation, migration, and invasion (Figure 5, H–J). These findings revealed that SETD1A regulated HCC stemness and progression partly via PABPC1. We then used CUT&Tag assay to determine the genomic distributions of SETD1A, H3K4me3, and PABPC1 in CD24^+^CD133^+^ liver CSCs and found that 54% (3,019/5,552) PABPC1 targets were co-occupied by SETD1A, 65% (3,604/5,552) PABPC1 targets were co-occupied by H3K4me3, 81% (6,511/8,004) SETD1A targets were co-occupied by H3K4me3, and 45% (2,499/5,552) PABPC1 targets were co-occupied by H3K4me3 and SETD1A (Figure 5K). In addition, GO analysis showed that PABPC1 targets were enriched for GO terms including protein phosphorylation, gene transcription, apoptosis, cell cycle, cell migration, and TGF-β signaling, all of which are involved in HCC stemness and progression (Figure 5L). GO analysis showed that co-occupied targets of SETD1A and PABPC1 as well as PABPC1, SETD1A, and H3K4me3 were enriched for protein phosphorylation, gene transcription, apoptosis, cell cycle, cell migration, and TGF-β signaling, indicating that SETD1A interacted with PABPC1, an interaction that is essential for the transcriptional activation of SETD1A-regulated oncogenes, to activate the co-regulated targets of SETD1A to promote HCC stemness and progression (Figure 5, M and N). Taken together, our data show that SETD1A cooperated with PABPC1 to regulate H3K4me3 modification on the promoters of oncogenes to drive HCC stemness.

## Discussion

In this study, we performed a genome-scale CRISPR knockout screening on CD24^+^CD133^+^ liver CSCs for determinants of HCC stemness and found that the most enriched were the genes associated with H3K4 methylation, chromatin remodeling, cell cycle, WNT, and noncanonical NF-κB pathway, especially the genes regulating H3K4 methylation, including the H3K4 methyltransferases KMT2A, KMT2D, and SETD1A. Among the above 3 H3K4 methyltransferases, SETD1A mainly catalyzes H3K4me3 at gene promoters and exhibits the most dramatic effect on global H3K4me3 and gene expression (40). Previous studies have indicated that increased H3K4me3 modification is associated with tumor progression and is a poor prognosis in patients with cancer (41). SETD1A has been found to promote tumors’ progression in various cancers, such as lung cancer, breast cancer, gastric cancer, colorectal cancer, and leukemia, by regulating TGF-β, Wnt, and Hippo/YAP signaling pathways (42). However, its role in CSCs remains unknown. Therefore, we focused on investigating the function of SETD1A in promoting HCC stemness. We showed that SETD1A expression is upregulated in HCC tissues and positively correlated with poor clinical outcome in patients with HCC, exhibiting good performance in predicting HCC relapse. SETD1A knockdown inhibited HCC stemness.

To further investigate the mechanism underlying SETD1A driving HCC stemness, we determined direct targets of SETD1A and its impact on histone modification profiling in liver CSCs. As a tagmentation-based epigenomic profiling method, CUT&Tag has distinct advantages compared with ChIP-Seq, such as easy handling, compatibility with reduced cell inputs, low costs, and high-quality data, therefore becoming the best fit in study of CSCs (43). Applying the CUT&Tag technique, we identified that SETD1A directly targets various histone-modifying enzymes in CD24^+^CD133^+^ liver CSCs, some of which have been reported to promote HCC stemness and progression. Histone modification profiling showed that SETD1A-generated H3K4me3 modifies the promoters of oncogenes involved in activation of WNT, MAPK, EGFR, and c-MYB pathways; autophagy; and protein kinase activity. In addition, we found that SETD1A promoted EZH2 transcription by directly binding to its promoter, leading to reprogramming of H3K27me3-modified genomic regions and inhibition of transcription of tumor suppressor genes related to cell proliferation suppression, differentiation, and collagen biosynthesis. Meanwhile, we found PABPC1 interacted with SETD1A/COMPASS to recruit SETD1A to the promoters of genes associated with protein phosphorylation, apoptosis, cell cycle, cell migration, and TGF-β signaling. These findings suggest that SETD1A serves as a master regulator for HCC stemness, representing an ideal target for HCC therapy.

Accumulating evidence has shown that epigenetic dysregulation contributes to aberrant transcriptional programs that promote tumor stemness and progression. Histone modification plays a central role in epigenetic regulation (44). Interestingly, we found that SETD1A directly binds to the promoters of various histone-modifying enzymes, which mediate H3K4me1, H3K4me3, H3K27me3, H3K9me3, H3K36me3, H3K79me3, and H4K16ac modification. Our study reveals that SETD1A drives HCC stemness through epigenetic regulation. These histone modifications and their crosstalk determine the transcriptional outcomes to promote HCC stemness and progression. Thus, further studies on SETD1A-mediated histone crosstalk are worthwhile to better understand the mechanism.

Our CRISPR screening also revealed that multiple members of TrxG proteins including KMT2A, KMT2D, SETD1A, SMARCE1, and SMARCD1 and PcG proteins including CBX4 and PCGF5 orchestrate HCC stemness, suggesting that TrxG and PcG proteins might cooperate or crosstalk to promote HCC stemness. TrxG and PcG proteins regulate cellular memory, cell fate determination, tumorigenesis, and tumor development via regulating histone modification, chromatin accessibility, and chromatin compaction (11). However, there are scarce reports regarding the role of TrxG and PcG proteins in HCC stemness. Among more than 40 family members of TrxG proteins, only BRG1 has been reported to promote HCC stemness through regulating lncRNA *lncFZD6* (45). Similarly, among PcG protein family members, BMI1 is the only one that has been reported to promote HCC stemness (46). Our study highlights the role of TrxG and PcG proteins in HCC stemness. Since a range of small molecules targeting TrxG and PcG proteins have gained success in preclinical development, such as WDR5-MLL interaction antagonist OICR-9429, MENIN inhibitor MI-403, and EZH2 inhibitor EPZ-6438 (47), our data provide the rationale to test these TrxG- or PcG-targeted drugs in the treatment of HCC.

An aberrant landscape of enhancers and SEs results in an abnormal transcriptional program, leading to tumorigenesis (48). SETD1A has been found to mediate long-range interactions between enhancer and promoter. Here, we demonstrate that SETD1A promotes activity of oncogenic enhancers and SEs in liver CSCs. It has been recently reported that H3K4me3 reader PHF23 interacts with SIN3-HDAC complex to mediate a synergistic action of H3K4me3 and H3K27ac on inhibition of the deacetylation activity of SIN3-HDAC complex, resulting in activation of tumor suppressor genes (30). This recent finding indicates that H3K4me3 methyltransferase, H3K27ac acetyltransferase, or H3K27ac deacetyltransferase might form a complex with H3K4me3 reader to mediate a synergistic effect of H3K4me3 and H3K27ac on regulation of gene transcription. The detailed mechanism underlying the regulatory effect of SETD1A on activity of oncogenic enhancers and SEs needs further investigation.

In summary, we identified the H3K4 methyltransferase SETD1A as an oncogenic regulator driving HCC stemness through epigenetic modification and PABPC1 to promote oncogene transcription, providing an attractive therapeutic target for the treatment of HCC.

## Methods

### Cell lines and cell culture.

HEK293T cells and HCC cell lines PLC, Huh7, and Hep3B were purchased from Cell Bank of the Chinese Academy of Sciences (Shanghai, China). HCC cells were cultured in DMEM high glucose (HyClone) supplemented with 10% fetal bovine serum (WISENT), 50 U/mL penicillin, and 50 mg/mL streptomycin (HyClone). All the cell lines were authenticated by short tandem repeat profiling and proved to be Mycoplasma-free by Myco-Blue Mycoplasma Detector (Vazyme).

### Antibodies and reagents.

Antibodies used were APC-conjugated CD133 antibody (catalog 130-113-184) from Miltenyi Biotec; PE-conjugated anti-CD24 (catalog 555428) from BD Biosciences; FITC-conjugated anti-EpCAM (catalog 60136FI) from StemCell Technologies; anti-SETD1A (catalog A300-289A) from Bethyl Laboratories; anti-PABPC1 (catalog A14872), anti-WDR5 (catalog A3259), anti-CXXC1 (catalog A13423), and anti-ASH2L (catalog A4892) from Abclonal; anti-H3K27ac (catalog ab4729) and anti-H3K27me3 (catalog ab6002) from Abcam; anti–β-actin (catalog AC004) from Abclonal Technology; anti–E-cadherin (catalog 3195S), anti-Vimentin (catalog 5741S), and anti-H3K4me3 (catalog C42D8) from Cell Signaling Technology; and Alexa Fluor Plus 488–conjugated goat anti-rabbit IgG (catalog A32731) and Alexa Fluor 594–conjugated donkey anti-rabbit IgG (catalog A-21207) secondary antibodies from Thermo Fisher Scientific. The detailed information for antibodies is shown in Supplemental Table 3. DAPI (catalog D9542) was from MilliporeSigma. B27 (catalog A3582801) and N2 supplements (catalog 17502001) were from Gibco. bFGF (catalog 157AA) was from Novoprotein, and EGF (catalog 236-EG) was from R&D Systems.

### Genome-wide CRISPR/Cas9 knockout library screen.

Human CRISPR Knockout Pooled Library (GeCKO V2, 1000000049) was purchased from Addgene. The libraries were amplified using Endura cells (catalog 60242, Lucigen). The workflow of CRISPR/Cas9 pooled screen is shown in Figure 1A. PLC cells were infected with lentiviral particles packaging Cas9 protein at an MOI less than 0.7. The blasticidin-selected Cas9-expressing PLC cells were infected with pooled lentiviral CRISPR library at an MOI of 0.3 (1,000× coverage) to ensure single-copy sgRNA integration in each cell. A pool of knockout cells was created after 7 days of selection with 2.5 μg/mL puromycin. CD24^+^CD133^+^ HCC CSCs and CD24^–^CD133^–^ non-HCC CSCs were sorted by FACS. Genomic DNA of cells were extracted using Quick-DNA Microprep Plus Kit (catalog D4074, Zymo Research) according to the manufacturer’s instructions. Sequencing libraries were constructed and sequenced by GENEWIZ company. The sequencing data were analyzed by MAGeCKFlute (49).

### Establishment of SETD1A-knockdown cell lines.

shRNA targeting SETD1A was cloned into pLKO.1-puro vector. The sequence is shown in Supplemental Table 1. To generate stable SETD1A-knockdown cell lines, lentiviral particles were generated by co-transfection of pLKO.1-shSETD1A, the packaging plasmid psPAX2, and the envelope plasmid pMD2.G into HEK293T cells using the calcium phosphate transfection method and harvesting of the supernatant 48 hours and 72 hours after transfection to infect HCC cell lines. Then, the cells were selected by puromycin for 24 hours. The effect of SETD1A knockdown was evaluated by qRT-PCR and Western blotting analysis.

### RNA extraction and qRT-PCR.

Total RNA was extracted using NucleoZOL (Macherey-Nagel) according to the manufacturer’s instructions. cDNA was synthesized using HiScript III All-in-one RT SuperMix Perfect for qPCR kit (catalog R333-01, Vazyme). TB Green Premix Ex Taq (Tli RNase H Plus) (catalog RR420A, Takara) was used for qRT-PCR. To ensure the authenticity of the results, the experiments were performed in triplicate. Changes in mRNA expression were calculated based on comparison of the cycle threshold value after normalization to β-actin expression. The primers used in this study are listed in Supplemental Table 2.

### Western blotting.

Cell or tissue proteins were extracted by RIPA buffer (catalog P00013C, Beyotime) supplemented with cOmplete protease inhibitor cocktail (catalog 4693116001, MilliporeSigma) according to the manufacturer’s instructions. The protein concentration was determined by the Pierce BCA Protein Assay kit (catalog 23225, Thermo Fisher Scientific). We loaded 20 μg protein on the SDS-PAGE and transferred to a PVDF membrane, followed by immunodetection of proteins. β-Actin was used as a loading control.

### Cell proliferation and Transwell migration and invasion assay.

A live real-time IncuCyte ZOOM (IncuCyte S3, ESSEN Bioscience) was used to determine cell proliferation as previously described (50). In the Transwell migration and invasion assays, cells were suspended by serum-free medium and gently added in a Transwell chamber (catalog 3422, Corning) with or without Matrigel (catalog 356234, Corning). After incubating at 37°C for 24 hours, cells on the lower surface of the Transwell chamber were fixed, stained with 0.1% crystal violet (Solarbio), and counted at an original magnification of 200×.

### Sphere formation assay.

Cancer cells were seeded in Ultra-Low Attachment 6-well plates (catalog 3471, Corning) at a density of 1,500 cells/well and cultured in DMEM high-glucose medium supplemented with 1× B27, 1 × N2, 20 ng/mL EGF, and 20 ng/mL bFGF for 2 weeks. Spheres were counted at an original magnification of 40×.

### Immunohistochemistry.

Tissues microarrays (TMAs) with patients’ survival information were purchased from Shanghai Outdo Biotech Company (catalog HLivH180Su16). The TMAs were treated with citrate buffer at 95°C. Endogenous peroxidase was inactivated using 3% H_2_O_2_. The TMAs were blocked using goat serum for 1 hour at room temperature and then incubated with anti-SETD1A antibody overnight at 4°C. The TMAs were then incubated with HRP-conjugated secondary antibody (Supplemental Table 3) and detected using DAB. The nuclei were stained with hematoxylin.

### Mouse model.

Four-week-old female NOD/SCID mice were purchased from the Shanghai Model Organisms Center, Inc. Mice were housed in individual ventilated cage systems in a specific pathogen–free animal room and fed standard laboratory diet with water and food.

A total of 1 × 10^4^ or 5 × 10^4^ cancer cells mixed with Matrigel (catalog 356231, Corning) were injected subcutaneously into a NOD/SCID mouse. Each experimental group contained 8 mice. Tumor size was measured every 3 days. The animals were euthanized when tumor size reached 1.0–1.5 cm in diameter. The tumor-initiating frequency was calculated using Extreme Limiting Dilution Analysis (http://bioinf.wehi.edu.au/software/elda/).

### CUT&Tag.

CD24^+^CD133^+^ liver CSC and CD24^–^CD133^–^ non-CSC cells were sorted by the BD Aria Fusion Cell Sorter. CUT&Tag was performed using the Hyperactive In-Situ ChIP Library Prep Kit (catalog TD901-01, Vazyme) according to the manufacturer’s instructions. Briefly, a total of 100,000 cells were collected and washed using wash buffer supplemented with protease inhibitor cocktail. Then cells bound to ConA magnetic beads were suspended in the antibody dilution buffer and incubated with primary antibody at room temperature for 2 hours or 4°C overnight. ConA-bound cells were then washed to remove unbound primary antibody, resuspended in Dig-wash buffer containing secondary antibody (Supplemental Table 3), and incubated at room temperature for 2 hours. Samples were washed using Dig-wash buffer 3 times, resuspended in Dig-300 buffer containing Hyperactive pG-Tn5/pA Transposon, and incubated at room temperature for 2 hours. Samples were washed with Dig-300 buffer and resuspended in tagmentation buffer for 1 hour at 37°C. To stop tagmentation, 10 μL 0.5M EDTA, 3 μL 10% SDS, and 2.5 μL 20 mg/mL Proteinase K were added to samples and incubated for 1 hour at 55°C. The phenol-chloroform extraction method was used to extract DNA. To amplify libraries, PCR was performed using the following cycling conditions: 72°C, 3 minutes; 98°C, 30 seconds; 15 cycles of 98°C for 15 seconds, 60°C for 30 s, 72°C for 30 seconds, and 72°C for 5 minutes. The PCR-amplified sequencing library was further purified using 1.2× AMPure XP beads (catalog A63881, Beckman Coulter Life Sciences). The library quality control was prepared using Agilent 2200. DNA sequencing was performed using Illumina NovaSeq 6000.

### ATAC-Seq.

ATAC-Seq was performed using TruePrep DNA Library Prep Kit V2 for Illumina (catalog TD501, Vazyme). Briefly, a total of 50,000 cells were collected and washed by PBS buffer. Cells were centrifuged at 500*g* for 5 minutes at room temperature, resuspended in cold lysis buffer (10 mM Tris-HCl at pH 7.4, 10 mM NaCl, 3 mM MgCl_2_, 0.1% IGEPAL CA-630), and incubated on ice for 10 minutes. Nuclear pellets were then collected by centrifugation for 5 minutes at 500*g* at 4°C and resuspended in transposition reaction mix and incubated for 30 minutes at 37°C. DNA was extracted using 2× Agencourt AMPure XP beads. ATAC-Seq libraries were amplified using Phanta HS Super-Fidelity DNA Polymerase using the following the program: 72°C for 3 minutes; 98°C for 30 seconds; 15 cycles of 98°C for 15 seconds, 60°C for 30 seconds, 72°C for 30 seconds, and 72°C for 5 minutes. The PCR-amplified sequencing library was purified and sequenced as previously described.

### CUT&Tag and ATAC-Seq analysis.

For CUT&Tag and ATAC-Seq, the reads were aligned to hg19 using the Bowtie2 (version 2.3.4.3) and run with MACS2 to call peaks with a *q* value of less than 0.05 by using the MACS2 (version 2.1.2) with default parameters, a genome size of 2.7 × 10^–9^ bp, and the appropriate input control sample. Through annotatePeaks.pl in Homer (version 4.11), we further annotated the peaks with their related genes and distance to the closest transcription start sites (TSSs).

For H3K27ac pairwise comparison, reads from CUT&Tag were counted using featureCounts (version 2.0.1). To screen the differentially expressed genes (DEGs) between the groups, the data sets were analyzed using the R package DESeq2 (version 1.30.1) with the default DESeq2 settings. The values for statistical significance were set as adjusted *P* ≤ 0.05 and |fold-change| ≥ 1. Volcano maps were drawn using the R package ggplot2 (version 3.3.3). To functionally annotate DEGs, visualization and annotation of GO terms was performed by Metascape (http://metascape.org/gp/index.html#/main/step1). GSEA was performed using the GSEA software (version 4.1.0) from the Broad Institute. The default weighted enrichment method was applied for enrichment analysis. The random combination was set for 1,000 times.

### Identifying SEs.

To identify SEs, which were defined as regions of CUT&Tag enrichment for H3K27ac, we used the Rank Ordering of Super-Enhancers algorithm (https://bitbucket.org/young_computation/rose). The enhancer peaks of H3K27ac were stitched together if they were located within 12.5 kb of each other; peaks within 2.5 kb from a RefSeq TSS were excluded. To distinguish the SEs from the typical enhancers, the point along with *x* axis at which a line with a slope of 1 was tangent to the curve was found by scaling the data such that the *x* and *y* axes were from 0 to 1. Enhancers above this point were defined as SEs, while enhancers below that point were typical enhancers. Enhancers were then assigned to the transcript whose TSS was nearest the center of the enhancer.

### Co-IP.

PLC cells transduced with FLAG-tagged PABPC1 vector or empty vector were lysed using NP-40 buffer (150 mM NaCl, 1.5 mM MgCl_2_, 0.5% NP-40, 50 mM Tris-HCl at pH 8.0) supplemented with protease inhibitor cocktail (MilliporeSigma). Cell lysates were incubated with anti-FLAG antibody (catalog F1804, MilliporeSigma). Protein-antibody complexes were conjugated to Protein A Magnetic Beads (MedChemExpress) by incubation at 4°C overnight. The beads were washed with IP washing buffer 3 times. Proteins were dissolved in 1× SDS loading buffer by boiling. The interacted proteins were identified using MS.

### Statistics.

Statistical data were analyzed by SPSS version 13.0. Overall survival and disease-free survival curves were plotted by Kaplan-Meier survival analysis and log-rank test. Two-tailed Student’s *t* test or 2-way ANOVA test was used to determine the differences among groups. *P* values less than 0.05 were considered statistically significant.

### Study approval.

All the animal experiments were approved by the Animal Ethics Committee of the Fifth Affiliated Hospital of Sun Yat-Sen University (Approval 2020101401) and were performed in compliance with the NIH guidelines.

### Data availability.

Values for all data points found in graphs are in the Supporting Data Values file. All the sequencing data have been deposited at National Center for Biotechnology Information Sequence Read Archive (accession number PRJNA991165).

## Author contributions

FX and HS were responsible for conceptualization. FX, JC, HH, ZX, and YT were responsible for methodology. ZX was responsible for bioinformation analysis. FX and JC were responsible for writing.

## Supplementary Material

Supplemental data

Supporting data values

## Figures and Tables

**Table 1:**
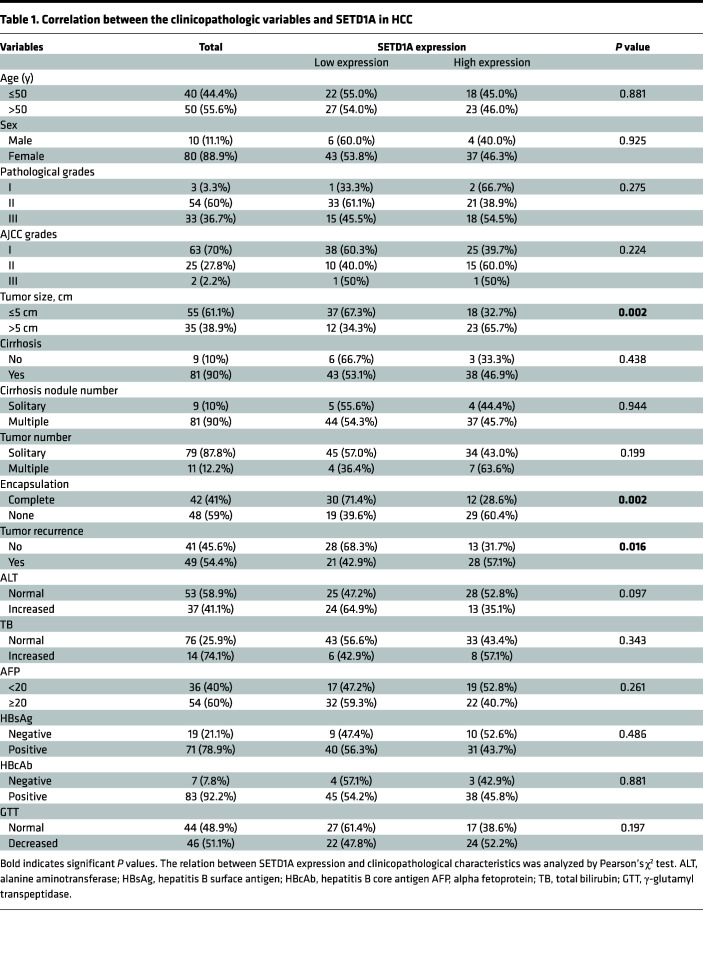
Correlation between the clinicopathologic variables and SETD1A in HCC

**Table 2:**
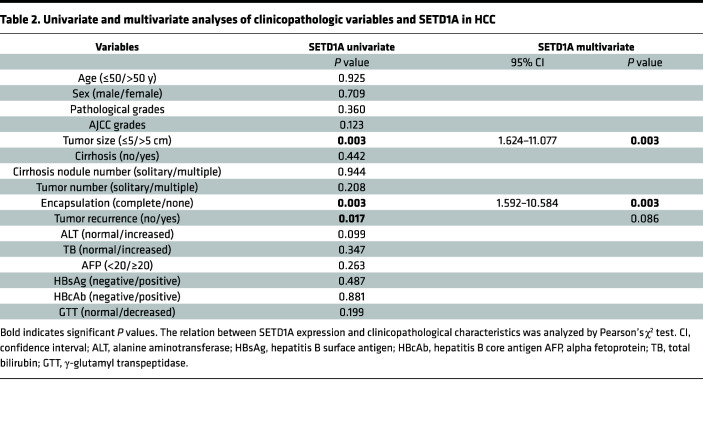
Univariate and multivariate analyses of clinicopathologic variables and SETD1A in HCC

## Version 1 08/15/2023

In-Press Preview

## Version 2 09/22/2023

Electronic publication
