# Supplementary material for: SETD1A drives stemness by reprogramming the epigenetic landscape in hepatocellular carcinoma stem cells
Source: JCI Insight. 2023 Sep 22;8(18):e168375. doi: 10.1172/jci.insight.168375 (PMC10561725; doi:10.1172/jci.insight.168375)
Supplement: Supplemental data [file jciinsight-8-168375-s041.pdf]

Supplemental Materials

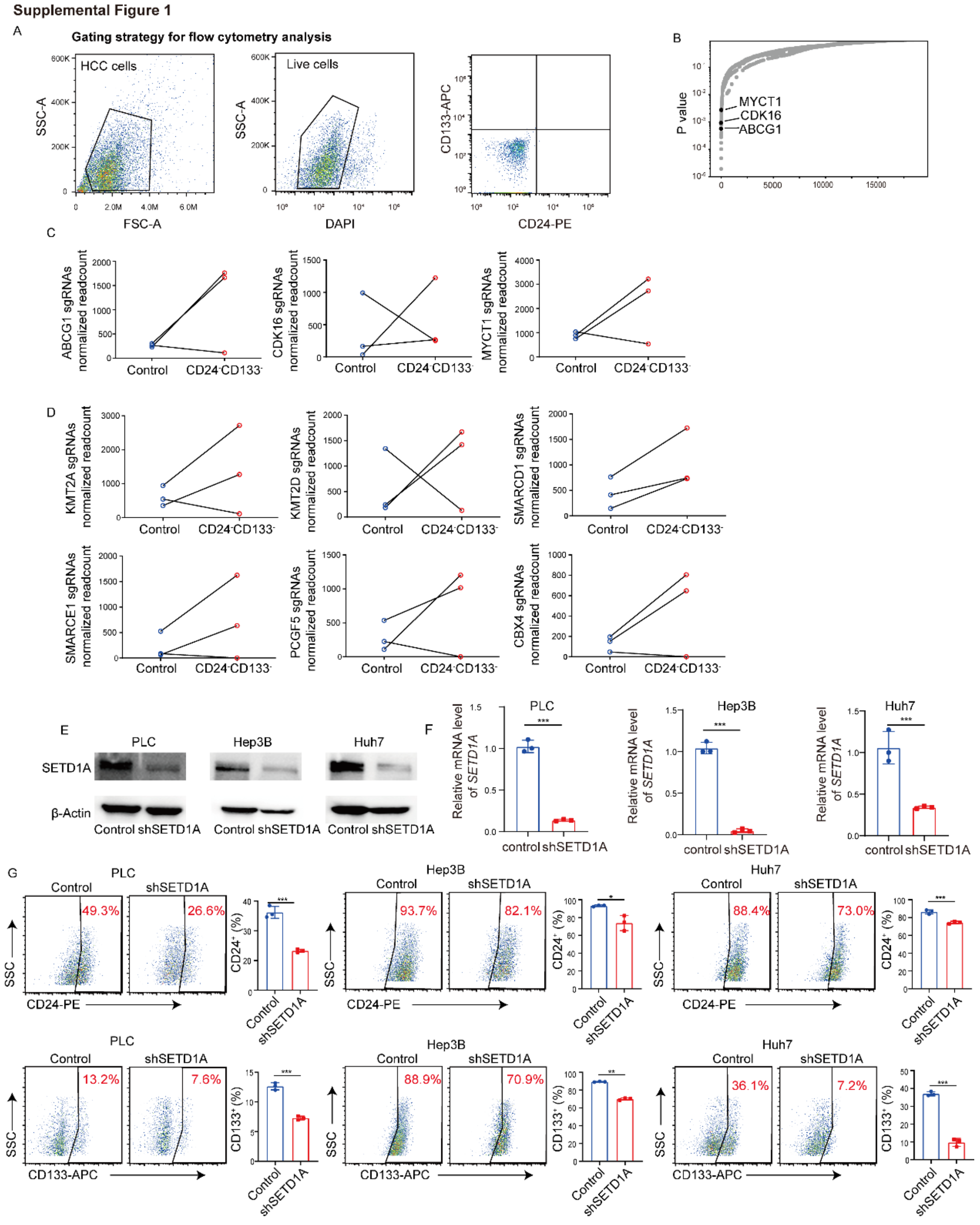

Supplemental Fig. 1. The CRISPR/Cas9 knockout screen for the key genes for CD24$^+$CD133$^+$ liver CSCs expansion. A. A scatterplot of the gRNA distribution from the

GeCKO screen. Known liver CSCs positive genes are shown. **B.** Enrichment of the gRNAs targeting ABCG1, CDK16, and MYCT1 gRNAs in the $CD24^{-}CD133^{-}$ non-CSCs. **C.** Enrichment of the gRNAs targeting KMT2A, KMT2D, SMARCD1, SMARCE1, PCGF5 and CBX4 in the $CD24^{-}CD133^{-}$ non-CSCs. **D.** Western blotting analysis of SETD1A expression in the control and SETD1A knockdown HCC cell lines. β-Actin was used as the loading control. **E.** qRT-PCR analysis of SETD1A expression in the control and SETD1A knockdown HCC cell lines (n=3). **F.** Flow cytometry analysis and quantification of $CD24^{+}$ and $CD133^{+}$ proportion in the scramble control and SETD1A knockdown HCC cells (n=3). Data are presented as mean ± SEM. Statistical analysis was performed by unpaired two-tailed Student's t-test. *p < 0.01, **p < 0.01, and ***p < 0.001.

Supplemental Figure 2

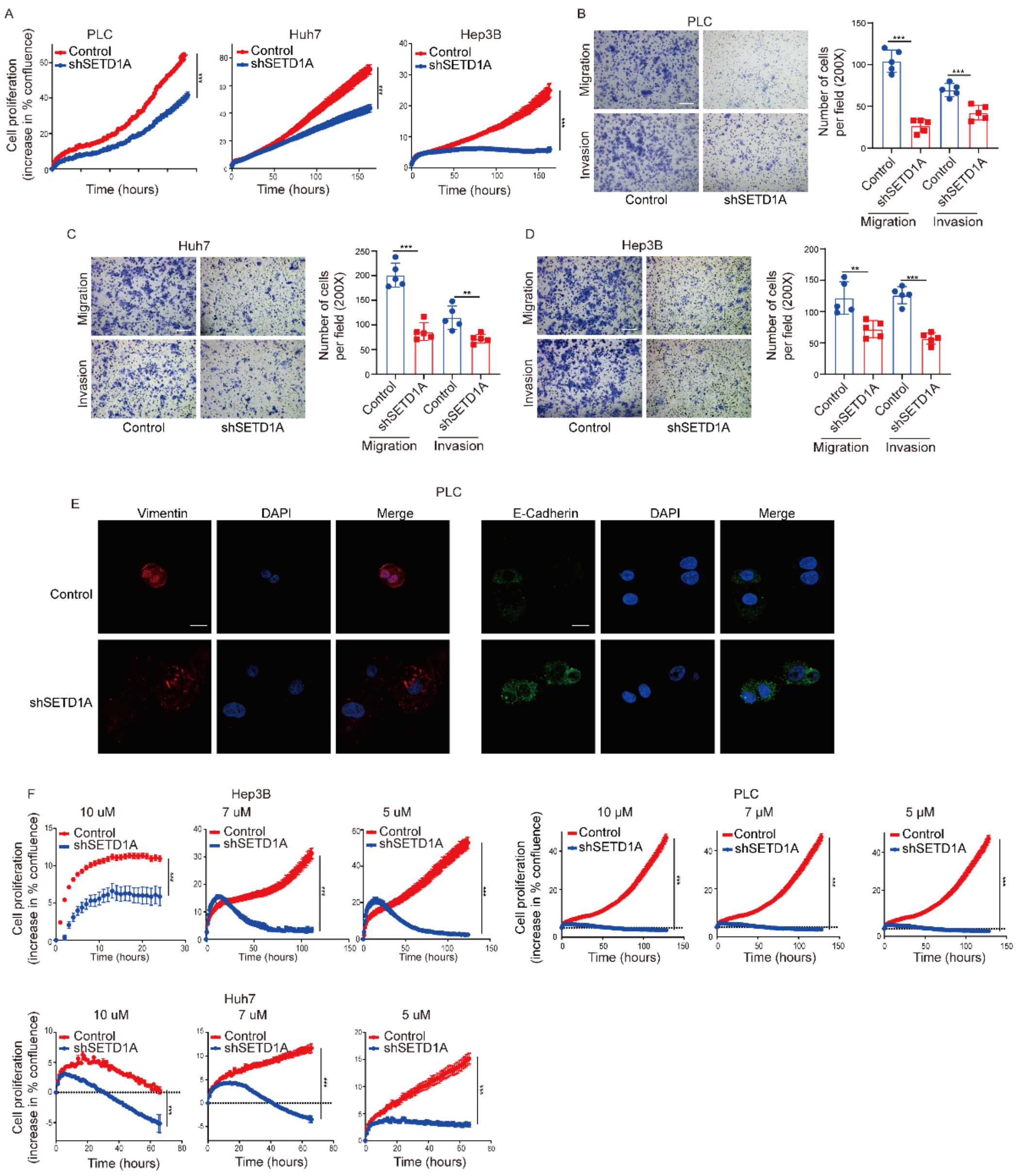

Supplemental Fig. 2. SETD1A promotes HCC proliferation, migration, invasion sorafenib

resistance and EMT. A. Cell proliferation assay for the effect of SETD1A knockdown on HCC cell proliferation (n=3). **B-D.** Transwell assay with/without Matrigel assessing the effect of SETD1A knockdown on HCC cell migration and invasion (n=3). Scale bar represents 200 μm. **E.** IF assay showing E-Cadherin and Vimentin expression upon SETD1A knockdown in HCC cells (n=3). Scale bar represents 200 μm. **F.** Cell proliferation assay showing that SETD1A leads to sorafenib resistance in HCC cells (n=3). Data are presented as mean ± SEM. Statistical analysis was performed by unpaired two-tailed Student's t-test. $^{**}p < 0.01$, and $^{***}p < 0.001$.

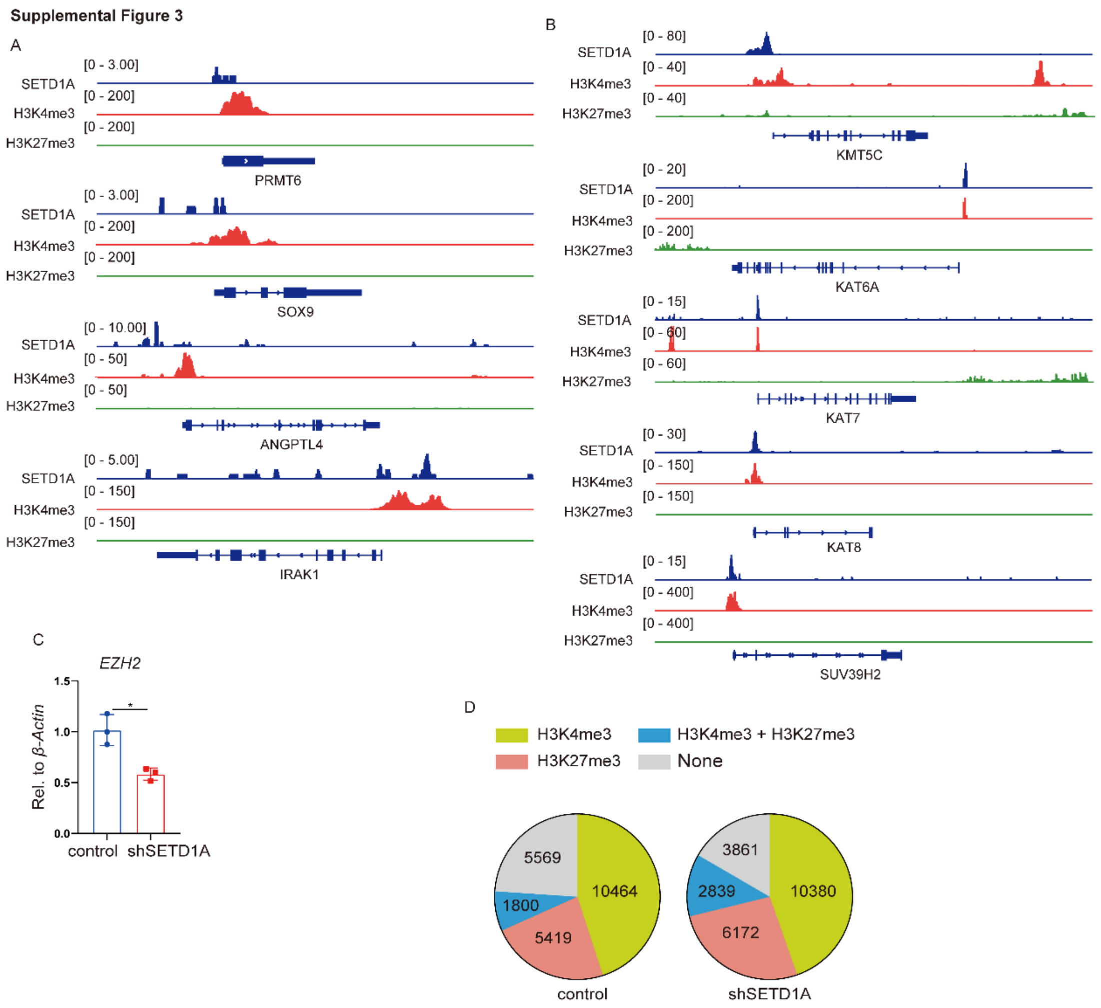

Supplemental Fig. 3. SEDT1A directly regulates transcription of various histone-modifying enzymes. A. Representative SETD1A CUT&Tag profiles in CD24^{+}CD133^{+} liver CSCs at *PRMT6*, *SOX9*, *ANGPTL4*, and *IRAK1* locus. **B.** Representative SETD1A CUT&Tag profiles in CD24^{+}CD133^{+} liver CSCs at *KMT5C*, *KAT6A*, *KAT7*, *KAT8*, and *SUV39H2* locus. **C.** qRT-PCR analysis of EZH2 expression in the control and SETD1A

knockdown CD24+CD133+ liver CSCs (n=3). **D.** Pie charts showing distribution of H3K4me3-marked genes, H3K27me3-marked genes, and bivalent genes in the control CD24+CD133+ liver CSCs and SETD1A knockdown CD24+CD133+ liver CSCs. Data are presented as mean ± SEM. Statistical analysis was performed by unpaired two-tailed Student's t-test. *$p < 0.05$.

Supplemental Figure 4

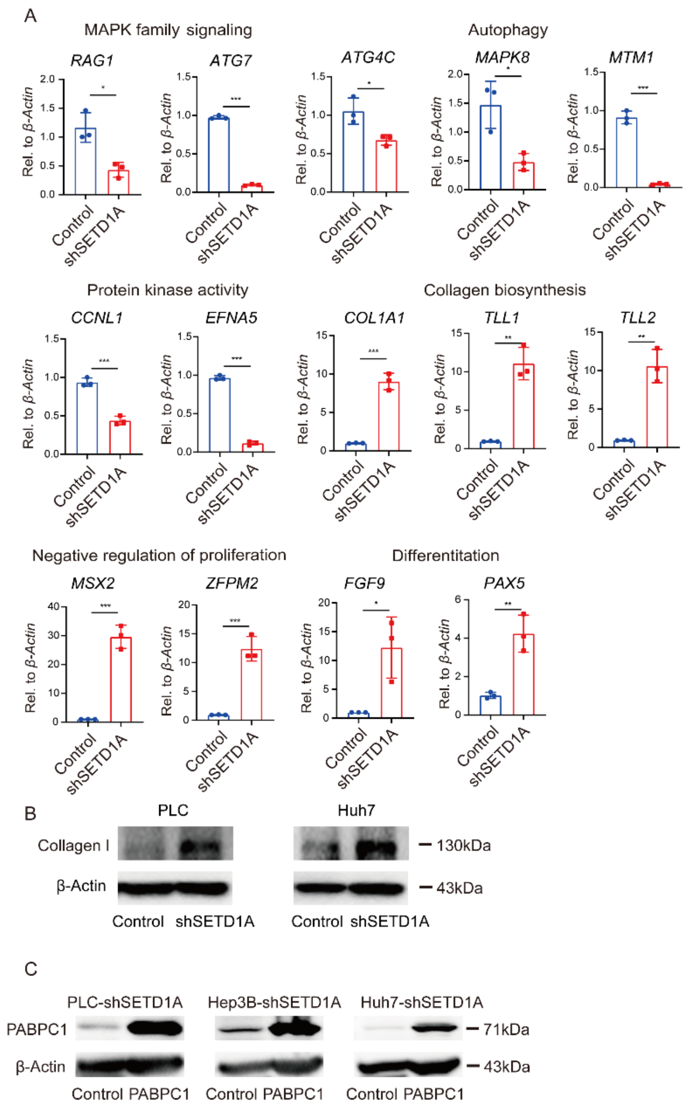

Supplemental Fig. 4. A. qRT-PCR analysis of representative genes associated with MAPK pathway, autophagy, protein kinase activity,

C. Western blot analysis of PABPC1 expression in SETD1A knockdown HCC cells

transduced with PABPC1 overexpression vector.

Supplemental Table 1. List of target sequence against SETD1A shRNA.

	Oligonucleotides (5' -> 3')
Target of shSETD1A	CAAAGGACAACAACGAATGAA

Supplemental Table 2. List of primer sequences used for qRT-PCR.

	Oligonucleotides (5' -> 3')
SETD1A Forward	CAGTGGCGGAACTACAAGCTC
SETD1A Reverse	CATAGCGGTACACCTTCTGAGA
KITLG Forward	TCTCGTTCAAGTTAATCCAGGTG
KITLG Reverse	ACATGGCTGAGGGGTAGCTT
EZH2 Forward	AATCAGAGTACATGCGACTGAGA
EZH2 Reverse	GCTGTATCCTTCGCTGTTTCC
WNT7A Forward	CTGTGGCTGCGACAAAGAGAA
WNT7A Reverse	GCCGTGGCACTTACATTCC
FNDC3B Forward	TCTCGTTCAAGTTAATCCAGGTG
FNDC3B Reverse	ACATGGCTGAGGGGTAGCTT
PTP4A1 Forward	GGCGTGGAGCTTTTAACAGC
PTP4A1 Reverse	GCCGCATTTTAGGACGATACTT
EPHA3 Forward	CTGCTCTGTTCTCGACAGCTT
EPHA3 Reverse	CAGCTCCCCTTGAATTGTTTTG
NGFR Forward	CCTACGGCTACTACCAGGATG
NGFR Reverse	CACACGGTGTTCTGCTTGT
AKAP12 Forward	GAGATGGCTACTAAGTCAGCGG
AKAP12 Reverse	CAGTGGGTTGTGTTAGCTCTTC
ST3GAL4 Forward	CTTCCTGCGGCTTGAGGATTA
ST3GAL4 Reverse	CTCACTCCCCTTGGTCCCATA
PTPN1 Forward	GCAGATCGACAAGTCCGGG
PTPN1 Reverse	GCCACTCTACATGGGAAGTCAC
ELF3 Forward	GGCCGATGACTTGGTACTGAC
ELF3 Reverse	GCTTGCGTCGTACTTGTTCTTC
β-Actin Forward	CATGTACGTTGCTATCCAGGC
β-Actin Reverse	CTCCTTAATGTCACGCACGAT

Supplemental Table 3. List of antibodies used in the study.

Name	Catalog number	Clone number	Reference (PMID)
APC-conjugated anti-CD133	cat. 130-113-184	293C3	10.1634/stemcells.2006-0258
PE-conjugated anti-CD24	cat. 555428	ML5	10.1186/bcr1610
FITC-conjugated anti-EpCAM	cat. 60136FI	VU-1D9	10.1172/JCI93707
Anti-SETD1A	cat. A300-289A		10.1074/jbc.M609809200
Anti-H3K27ac	cat. ab4729		10.1038/nature07829
Anti-H3K27me3	cat. ab6002	mAbcam 6002	10.1093/nar/gkl304
Anti-β-Actin	cat. AC004		10.1371/journal.ppat.1009616
Anti-E-cadherin	cat. 3195S	24E10	10.1002/mc.20531
Anti-Vimentin	cat. 5741S	D21H3	10.1042/BJ20050065
Anti-H3K4me3	cat. 9751	C42D8Lys4	10.1074/jbc.M103973200
Alexa Fluor Plus 488-conjugated Goat anti-Rabbit IgG	cat. A32731		10.3892/ijmm.2016.2817
Alexa Fluor 594-conjugated Donkey anti-Rabbit IgG	cat. A-21207		10.1186/1743-422X-8-461
